# Depression of Family Caregivers Is Associated with Disagreements on Life-Sustaining Preferences for Treating Patients with Dementia

**DOI:** 10.1371/journal.pone.0133711

**Published:** 2015-07-31

**Authors:** Chia-Fen Tsai, Yao-Tung Lee, Wei-Ju Lee, Jen-Ping Hwang, Shuu-Jiun Wang, Jong-Ling Fuh

**Affiliations:** 1 Department of Psychiatry, Neurological Institute, Veterans General Hospital, Taipei, Taiwan; 2 Department of Neurology, Neurological Institute, Taipei Veterans General Hospital, Taipei, Taiwan; 3 Institute of Brain Science, National Yang-Ming University Schools of Medicine, Taipei, Taiwan; 4 Institute of Clinical Medicine, National Yang-Ming University Schools of Medicine, Taipei, Taiwan; 5 Faculty of Medicine, National Yang-Ming University Schools of Medicine, Taipei, Taiwan; 6 Department of Neurology, Taichung Veterans General Hospital, Taichung, Taiwan; 7 Department of Psychiatry, Taipei Medical University–Shuang Ho Hospital, New Taipei City, Taiwan; "Mario Negri" Institute for Pharmacological Research, ITALY

## Abstract

**Background:**

Family caregivers may not agree with patients with dementia regarding attitudes toward end-of-life preferences, and the effects of this type of disagreement are not well understood. This study sought to identify such a disagreement and its predictors.

**Methods:**

A cross-sectional sample of 84 family caregivers and patients with dementia was recruited from memory clinics. We used the Mini-Mental State Examination, Neuropsychiatric Inventory, Clinical Dementia Rating, and Katz index of independence in activities of daily living to assess patient symptoms, functions, and severity of dementia. Caregivers completed questionnaires on perceived patient end-of-life care preferences, caregiver end-of-life care preferences for patients, Zarit Burden Interview (ZBI), Center for Epidemiological Studies–Depression Scale (CES-D), and knowledge of clinical complications of advanced dementia.

**Results:**

The self-disclosure rates of patient preferences were 34.5% for tube feeding, 39.3% for cardiopulmonary resuscitation, and 45.2% for mechanical ventilation. For patients who had disclosed preferences, the disagreement rate between them and their caregivers was 48.3% for tube feeding, 48.5% for cardiopulmonary resuscitation, and 60.3% for mechanical ventilation. Caregiver depression (i.e., CES-D ≥16) was associated with disagreements on cardiopulmonary resuscitation (adjusted odds ratio (aOR) = 6.6, 95% CI = 1.4–31.1, *P* = 0.01) and mechanical ventilation (aOR = 14, 95% CI = 2.2–87.2, *P* = 0.005) preferences.

**Conclusion:**

The preferences of end-of-life issues differed greatly between dementia patients and their caregivers. Depression in caregivers is associated with such discrepancy.

## Introduction

In Taiwan, prevalence of dementia is 5% among adults aged above 65 years and the number of people with dementia is expected to double every 20 years.[1] Dementia is a progressive degenerative disease and is a predictor of death with two to three times the risk of other illnesses in elderly people aged 65 years or older.[2] Studies have suggested that patients with advanced dementia are under-recognized as being at high risk of death and receive suboptimal palliative care.[3] Decisions to receive palliative treatment instead of life-sustaining treatment can be difficult, because most patients with dementia are unable to make these decisions for themselves. Therefore, relatives, nurses, and physicians must occasionally make these decisions for a demented patient.

To fulfill the best interests of the patient, family caregivers must adequately understand the preferences of the patient. However, the results of studies that have evaluated the end-of-life decision preferences of patients and family caregivers are inconsistent. Studies have found that approximately one-third of caregivers inaccurately predicted patient preferences,[4] and caregivers tended to project their own preferences, rather than truly state patient preferences.[5] A recent study found that caregivers of patients with dementia increasingly deemphasize the importance of values over time, and may become increasingly unable to make decisions that effectively represent patient preferences.[6] Therefore, surrogate decision makers for patients with dementia may disagree with patient preferences. This may result in patients with dementia who have not documented their treatment preferences through an advanced directive being vulnerable to receiving unwanted interventions at the end of their lives. An examination of the different values of family members concerning life-sustaining treatment has not been conducted in patients with dementia in Asian countries, and further research focusing on the influence of disagreement is necessary.

A clearer understanding of factors related to preference disagreements may provide health care providers with more information for assisting patients and families when making medical decisions, and may improve the quality of end-of-life care for patients with advanced dementia. The purposes of this study were (1) to examine disagreements on life-sustaining treatment preferences among family caregivers; and (2) to explore the factors that influence these preferences and disagreements, and to examine family caregiver preferences when they reported that patients have not disclosed those preferences.

## Method

### Participants

We recruited patients with dementia and their family caregivers from the Memory Clinic of Taipei Veterans General Hospital. The diagnosis of dementia was based on the criteria of the Diagnostic and Statistical Manual of Mental Disorders, 4th edition, text revision,[7] and diagnosing Alzheimer’s dementia (AD) was based on the criteria of the National Institute of Neurological and Communicative Disorders and Stroke and the Alzheimer’s Disease and Related Disorders Association (NINCDS-ADRDA).[8]

We evaluated the general cognitive functions, neuropsychiatry symptoms, independence in activities of daily living, and dementia severity of the patients for investigating caregiver burdens, knowledge of the clinical complications of advanced dementia, and mood statuses. The general demographic information that was collected included age, sex, years of education, time since dementia diagnosis, dementia subtype, nursing home residence, and religious beliefs. The study protocol was reviewed and approved by the institutional review board of Taipei Veterans General Hospital. Written informed consent was obtained from the patients or their legally authorized representatives, and from family caregivers. A caregiver was defined as someone who spent a minimum of 4 hours per week caring for the person with dementia. This study included only informal family caregivers.

### Measurement

#### Life-sustaining treatment preferences

The family caregivers completed a questionnaire to provide their perceptions of life-sustaining treatment preferences of patients. (Did the patient disclose their preference to you? If the answer is yes, please report the perceived preferences from demented patient and also report your preferences of life-sustaining treatment for demented patient. If the answer is no, please report your preferences of life-sustaining treatment for demented patient). The preferences are simplified to three options (“conduct life-sustaining treatment,” “no life-sustaining treatment,” or “unsure”). The decisions made for patients regarding three life-sustaining treatments were tube feeding, cardiopulmonary resuscitation, and mechanical ventilation. Family caregivers of patients with undisclosed preferences were defined as surrogate decision makers in the present study. Disagreement of preference was referred, whereas family caregivers’ preference differed from patients’ preference.

#### Mini-mental state examination

The mini-mental state examination (MMSE) is one of the most widely used screening instruments for dementia and provides a total score ranging from 0 to 30, with lower scores indicating greater cognitive impairment. It is administered to patients to obtain the overall level of their current cognitive functions.[9]

#### Clinical dementia rating scale

The clinical dementia rating (CDR) scale is designed to rate dementia severity on a five-point scale, based on the patient’s performance in six areas of cognition and daily living.[10] The CDR was administered during structured interviews with patients and their caregivers, and a physician rated patient performance in each area to generate a total CDR score. The total CDR score ranges from 0 to 3, where 0 = *normal*, 0.5 = *very mild dementia*, 1 = *mild dementia*, 2 = *moderate dementia*, and 3 = *severe dementia*.

#### Katz index of independence in activities of daily living

The ability of the participant to perform activities of daily living independently was assessed using the Katz index of independence in activities of daily living (KI), which was designed for assessing older adults and has been applied to numerous populations.[11] The index ranks performance adequacy for six functions: bathing, dressing, toileting, transferring, continence, and feeding. Patients score “yes” or “no” for independence in each of the six functions. A score of 6 indicates full function.

#### Neuropsychiatric inventory

The neuropsychiatric inventory (NPI), a measure of the frequency and severity of 12 psychiatric symptoms (delusions, hallucinations, agitation, depression, anxiety, euphoria, apathy, disinhibition, irritability, abnormal motor output, appetite, and sleep), was used to assess patient behavioral symptoms during the previous month.[12] The NPI was administered during structured interviews, in which family caregivers rated the frequency (on a scale of 1–4) and severity (on a scale of 1–3) of each symptom that received a positive response, based on screening questions for each behavior. The NPI symptom score is calculated by multiplying the severity and frequency scores, yielding a score between 1 and 12 (inclusive) if a symptom is present, and a score of 0 if a symptom is absent.

#### Zarit burden interview

The Zarit burden interview (ZBI) is a self-administered, 22-item instrument that measures caregiver perceptions of the burden of providing care. The questionnaire addresses areas that caregivers commonly report as problematic, such as physical health, psychological well-being, finances, and their relationship with the patient. Responses to each item are structured on a five-point Likert scale ranging from 0 (*never*) to 4 (*nearly always*), with a total possible score of 0 to 88. Higher scores indicate an increased caregiver burden.[13]

#### Center for Epidemiological Studies–Depression Scale

Caregiver depressive symptoms were evaluated using the self-administered Center for Epidemiological Studies–Depression Scale (CES-D),[14] which was designed to measure depressive symptoms in nonpsychiatric participants. Responses to each of the 20 items are structured on a four-point scale based on the frequency of the symptom during the preceding week. A total score above 16 indicates a high risk of clinical depression. We used the CES-D because of its relatively high internal consistency (Cronbach’s *α* = 0.87) and predictive validity for diagnosing depression in family caregivers. A CES-D cutoff score of 16 indicates symptomatic depression.

#### Caregiver Knowledge of Clinical Complications of Advanced Dementia

To assess caregiver knowledge of the clinical complications of advanced dementia, we designed a questionnaire based on a recent publication that focuses on this concern.[15] Caregivers were asked to independently complete this questionnaire after the interview. It consisted of 3 symptoms: febrile episodes, pneumonia, and eating problems. The items involved using a true/false format, with each correct answer resulting in a point added to the total score (0–3 points). A higher total score indicates greater factual knowledge.

### Statistical analyses

Statistical analyses were performed using SPSS software (version 18.0; IBM Inc., Armonk, NY, USA). Descriptive statistics are expressed as means ± standard deviations. For categorical data, the chi-square test or Fisher’s exact tests were used to test the difference between groups. The Student’s *t*-test was used to compare the means of continuous variables. In addition to the percentage of overall agreement, kappa coefficients were computed to assess the extent of concordance between perceived end-of-life care preferences of patients and caregivers to correct or adjust for the amount of agreement that is expected to occur by chance.[16] We followed criteria proposed by Landis and Koch[17] for kappa as a measure of the strength of agreement: ≤0.2, poor; 0.21 to 0.4, slight; 0.41 to 0.6, moderate; 0.61 to 0.8, substantial; and 0.81 to 1, nearly perfect.

We used logistic regression analysis to investigate the predictors that contribute to family preferences of no life-sustaining treatment among patients without self-disclosure preferences and the preference disagreement rate between patients and their family caregivers. Based on the univariate analysis results, we incorporated four independent variables into the logistic regression model of preferences (knowledge of advanced dementia complications score, MMSE score, CDR score, and patient age) and three variables for the disagreement rate (ZBI results, symptomatic depression, and disease duration). Bivariate analyses were performed using the Pearson correlation to identify the factors influencing the preference for less invasive treatment among family caregivers who disagreed with their patients. The statistical significance level was set at *P* < 0.05.

## Results

### Participants

During the study period, we enrolled 84 patients with dementia, of whom 67 (79.8%) had a diagnosis of probable or possible AD, 12 (14.3%) had Lewy body dementia, 4 (4.8%) had vascular dementia, and 1 (1.2%) had semantic dementia. Table 1 lists the demographic and clinical characteristics of the patients and their caregivers. The mean age of the patients was 82.7 ± 7 years. More than half of the patients (N = 47, 56%) were female. Most of the patients were married (76.2%) and lived with their families (88.1%). Most of the patients had mild to moderate dementia and were residing at home (96.4%). The mean length of time since diagnosis was 4.1 ± 3.2 years. Most family caregivers were younger than 65 years old (69%) and had high educational levels (≥16 years of education, 62%). More than half of the caregivers (N = 54, 64%) were female and married (N = 64, 76.2%). Most of the family caregivers were middle-aged offspring (spouses, 25%; daughters, 33.3%; sons, 23.8%; daughters-in-law, 9.5%; grandchildren, 2.4%; and other relatives, 6%).

**Table 1:** Demographic and clinical characteristics of the patients and their caregivers.

	Patients (N = 84)	Family caregiver (N = 84)
	Mean ± S.D/ Number (%)	Mean ± S.D/ Number (%)
**Age**	82.7±7.0 (range 68–90)	51.8±12.9 (range 20–80)
**Gender (Female)**	47 (56.0%)	54(64.3%)
**Current married**	64 (76.2%)	64(76.2%)
**Educational level**		
0–9 years	42 (50%)	14(16.7%)
12 years	22 (26.2%)	17(20.2%)
≧16 years	20 (23.8%)	53 (63.1%)
**MMSE**	17.1±6.2 (range 5–27)	
**CDR**		
0.5	1 (1.2%)	
1	50 (59.5%)	
2	24 (28.6%)	
3	9 (10.7%)	
**NPI**	31.6±36.7 (range 0–130)	
**Katz Index (KI)**	2.8±2.4(range 0–6)	
**Duration after diagnosis (years)**	4.1±3.2(range 0.5–13)	-
**No religious belief**	28 (33.3%)	-
**Nursing home residence**	3 (3.6%)	-
**Knowledge score (KC)**		
**0**		27(32.1%)
**1**		32(38.1%)
**2**		15(17.9%)
**3**		10(11.9%)
**ZBI**	-	33.0±16.4(range 2–77)
**CES-D**	-	14.4±10.3(range 0–44)

SD: Standard deviation;The Mini-Mental State Examination (MMSE); Clinical Dementia Rating (CDR); Neuropsychiatric Inventory (NPI); Zarit Burden Interview (ZBI); Center for Epidemiological Studies–Depression Scale (CES-D); Katz index of independence in activities of daily living (KI); knowledge of clinical complication of advanced dementia (KC)

### Family caregiver preferences for patients with undisclosed preferences

Table 2 lists family caregiver preferences for end-of-life care among patients without self-disclosed preferences. The rate of undecided caregiver preferences for patients without self-disclosure was higher than that for patients with self-disclosure (49.1% vs. 37.9% for tube feeding; 47.1% vs. 42.4% for cardiopulmonary resuscitation; 39.1% vs. 37.8% for mechanical ventilation). In the no tube feeding group, the patients had lower MMSE scores (10.8 ± 6.1 vs.17.3 ± 5.5, *P* = 0.02) than those of patients with tube feeding preferences. In the no cardiopulmonary resuscitation group, family caregiver mean KC scores were higher (1.8 ± 1 vs. 0.9 ± 0.9, *P* = 0.003), patients were older (89.1 ± 6 vs. 82.3 ± 6.3, *P* = 0.001), and patient MMSE scores were lower (13.1 ± 6.9 vs. 17.5 ± 5.9, *P* = 0.03). In the no mechanical ventilation group, the caregiver KC scores were higher (2 ± 0.9 vs. 0.8 ± 0.8, *P* < 0.001) and the patients were older (86.5 ± 5.9 vs. 81.9 ± 6.5, *P* = 0.02). Binary logistic regression analysis (Table 3) indicated that the family caregivers’ KC scores of advanced dementia complications were significantly associated with preferences for no cardiopulmonary resuscitation (OR = 2.5, 95% CI = 1–6, *P* = 0.04, adjusted with patient age and MMSE) and no mechanical ventilation (OR = 5.1, 95% CI = 2–13.2, *P* = 0.001, adjusted with patient age). A lower MMSE score was associated with a preference for no tube feeding (OR = 0.8, 95% CI = 0.7–0.9, *P* = 0.01). A spousal relationship was not associated with the incidence of disagreements. Between patients with and without self-disclosure, no significant difference in the proportion of undecided caregiver preferences for tube feeding (OR = 0.63, 95% CI = 0.25–1.59, *P* = 0.23), cardiopulmonary resuscitation (OR = 0.83, 95% CI = 0.34–2.00, *P* = 0.42), or mechanical ventilation (OR = 0.91, 95% CI = 0.37–2.20, *P* = 0.51) was found.

**Table 2:** Preferences for life-sustaining treatments reported by patients and their family caregivers.

Patient’s attitude (N = 84)	Never disclosure	Self disclosure			
	Families’ preference	Patients’ preference	Families’ preference	Disagreement rate	Kappa
**Tube feeding**				48.3%	0.30
**Yes**	19(34.5%)	7(24.1%)	11(37.9%)		
**No**	9 (16.3%)	14(48.3%)	7(24.1%)		
**Indecisive**	27(49.1%)	8(27.6%)	11(37.9%)		
**Cardiopulmonary resuscitation**				48.5%	0.29
**Yes**	11(21.6%)	6 (18.2%)	10(30.3%)		
**No**	16(31.4%)	19 (57.6%)	9 (27.3%)		
**Indecisive**	24(47.1%)	8 (24.2%)	14(42.4%)		
**Mechanical ventilation**				60.3%	0.30
**Yes**	7(15.2%)	4(10.5%)	5(13.5%)		
**No**	21(45.7%)	27(71.1%)	18(48.6%)		
**Indecisive**	18(39.1%)	7(18.4%)	14(37.8%)		

**Table 3:** Multiple logistic regression models of (a) disagreement of the life-sustaining treatment preference among patients with self disclosure and (b) family caregivers’ preference for patient among patients without self disclosure.

**(a)Patients with self disclosure**	**Disagreement**					
	**Tube feeding**		**Cardiopulmonary resuscitation**		**Mechanical ventilation**	
	OR(95% CI)	P-value	OR(95% CI)	P-value	OR(95% CI)	P-value
Depression	**-**	-	6.6(1.4–31.1)^a^	0.01	14.0(2.2–87.2)^b^	0.005
**(b)Patients without disclosure**	**Family caregivers’ preference for patients**					
	**No Tube feeding**		**No Cardiopulmonary resuscitation**		**No Mechanical ventilation**	
	OR(95% CI)	P-value	OR(95% CI)	P-value	OR(95% CI)	P-value
Knowledge of advanced dementia	**-**	-	2.5(1.0–6.0)^c^	0.04	5.1(2.0–13.2)^d^	0.001
MMSE	0.8(0.7–0.9)	0.01	-	-	-	-

a: adjusted with ZBI

b: adjusted with disease duration

c: adjusted with patient’s age and MMSE

d: adjusted with patient’s age

Depression: defined by the score of Center for Epidemiological Studies–Depression Scale (CES-D)≧16. OR: odd ratio. CI: confidence interval. MMSE: The Mini-Mental State Examination; CDR: Clinical Dementia Rating; Zarit Burden Interview (ZBI).

### Patient disclosure of life-sustaining treatment preferences

Table 2 lists patient and family caregiver life-sustaining treatment preferences and patient disclosure of these preferences. Fewer than half of the patients (35%–45%) had disclosed their preferences toward end-of-life decisions to family caregivers. The patient preference rate for life-sustaining treatment was 24.1% for tube feeding, 18.2% for cardiopulmonary resuscitation, and 10.5% for mechanical ventilation. However, approximately one-fourth of the patients were hesitant to make a decision.

Patients with dementia who had expressed self-disclosure preferences for tube feeding and cardiopulmonary resuscitation were younger than those who had not (80.6 ± 7.1 vs. 83.9 ± 6.7, *P* = 0.04; 80.1 ± 6.2 vs. 84.4 ± 6.9, *P* = 0.004). The severity of dementia was milder (CDR = 0.5 or 1 vs. CDR = 2 or 3) for those who chose cardiopulmonary resuscitation (*P* = 0.01) and mechanical ventilation (*P* = 0.01). No significant differences were found for sex, educational level, MMSE score, Katz index, disease duration, or religious beliefs.

### Disclosed end-of-life care preference disagreements between patients with dementia and their family caregivers

The disagreement rate for life-sustaining treatment preferences of patient-caregiver pairs was 48.3% for tube feeding, 48.5% for cardiopulmonary resuscitation, and 60.3% for mechanical ventilation. The kappa values for concordance between patient and family caregiver preferences was 0.3 for tube feeding, 0.29 for cardiopulmonary resuscitation, and 0.3 for mechanical ventilation, reflecting poor concordance between patients and their family caregivers on life-sustaining treatment preferences. Caregivers who disagreed with cardiopulmonary resuscitation preferences were more likely to have symptomatic depression (75% vs. 31.3%, *P* = 0.01) and had higher ZBI scores (43.8 ± 12.9 vs. 29.6 ± 17.4, *P* = 0.01) than did those who agreed with cardiopulmonary resuscitation preferences. Caregivers who disagreed with mechanical ventilation preferences were more likely to have symptomatic depression (78.6% vs. 34.8%, *P* = 0.01) and had longer diagnosis periods (6.5 ± 3.5 vs. 2.6 ± 2.5, *P* = 0.002) than did those who agreed with mechanical ventilation preferences.

Of the patient–caregiver pairs who disagreed, family caregivers preferred aggressive care (patients preferred no treatment vs. caregivers preferred life-sustaining treatment or depending preference) rather than invasive care (patients preferred life-sustaining treatment vs. caregivers preferred no life sustaining treatment or depending preference) for tube feeding (84.6% vs. 15.4%), cardiopulmonary resuscitation (81.3% vs. 18.7%), and mechanical ventilation (76.9% vs. 23.1%).

Significant correlations were also found among the patient-caregiver pairs who disagreed between higher NPI total scores and family caregivers’ preference for less invasive care for tube feeding (r = 0.54, *P* = 0.04); Lower CES-D score of family caregivers and family caregivers preferred less invasive care for cardiopulmonary resuscitation (r = 0.68, p = 0.01); Higher Katz index and family caregivers preferred less invasive care for mechanical ventilation (r = 0.54, p = 0.04)

Logistic regression analysis (Table 3) showed that family caregiver depression was independently associated with disagreements on cardiopulmonary resuscitation (OR = 6.6, 95% CI = 1.4–31.1, *P* = 0.01, adjusted with ZBI) and mechanical ventilation (OR = 14, 95% CI = 2.2–87.2, *P* = 0.005, adjusted with disease duration). Tube feeding disagreements and covariates were not significantly associated.

## Discussion

In this study, we assessed perceived patient and family own preferences, and preference disagreements regarding life-sustaining treatment. More than half of the family caregivers reported that the person with dementia had not disclosed their opinions or decisions on end-of-life situations to them. We found that family caregiver depression was associated with preference disagreements between patients and caregivers, and understanding the clinical complications of advanced dementia was related to family preferences for palliative care. When patient preferences differed from those of family caregivers, family caregivers tended to prefer life-sustaining treatment for patients.

The self-disclosed rate reported by family caregivers is low in the present data. The reasons for this may be complex. It is often reported that people with dementia are denied access to hospices.[18] The reasons for this are complex, but they may include the fact that dementia is not perceived as a terminal illness.[19] Most patients with advanced dementia have profound cognitive impairments and lack the capacity required to make decisions on their care and treatment, in contrast to those with cancer and other end-stage chronic diseases.[20]

The patients with dementia who had informed their families were more likely not to choose life-sustaining treatments; however, although they had expressed their preferences, the rate of consistent patient and family caregiver preferences was modest (concordance rate, 40%–52%; Kappa value, 0.29–0.3), and lower than the concordance rate in a study on terminally ill cancer patients in Taiwan (concordance rate, 62%–97%; Kappa value, 0.13–0.46).[21] Our data showed that symptomatic depression is significantly associated with a discrepancy between end-of-life treatment preferences. This association may be bi-directional. First, research has suggested that emotions can interfere with decision making,[22] and family caregivers of dementia patients experience high levels of psychological morbidity, depression, stress, and burdens.[23] Making decisions is also difficult when families are grieving, and caregivers of patients with dementia may experience anticipatory or predeath grief, because dementia often results in a loss of the self, long before physical death occurs.[24] Therefore, family members of patients with dementia who are prone to developing depressive symptoms may have reduced decision-making capacities. Physicians who care for people with dementia may consider evaluating family caregiver depression to help them make more integrated decisions. In the other hand, this disparity may increase caregivers’ stress and depression. In patients with cancer, disagreement within patient families can result in excessive stress and compromise the quality of life for patients and their family caregivers [25]

This study also showed that when preference disagreements exist, family caregivers prefer life-sustaining treatment over palliative care (73%–85%). Disagreement may influence the family caregivers’ preference for life-sustaining treatment. Previous research has shown that disagreements endanger the decision making and treatment choices of patients with cancer and their families.[26] Our results are similar to those of a report that focuses on spouses of people with dementia or mild cognitive impairment[27] and to the results of a study in Taiwan showing that family caregivers of terminally ill cancer patients were more likely to choose life-sustaining treatment than the patients themselves.[21] This may result in patient preferences being overridden at the end of life. In assessing the factors influencing preferences for palliative care at the end of life, our study demonstrated the importance of caregivers’ awareness of their own emotional status and the underlying disease statuses of patients, including the severity of the patients’ behavioral and psychological symptoms of dementia and functional performance. Previous studies have reported that current health is related to end-of-life decision making in patients referred to palliative care consultation or community-dwelling elderly. [28,29]

In this study, of the patients with uncertain preferences, approximately half of their family caregivers also chose the option of “indecisiveness”. This reflects family caregiver hesitation, which may occur because caregivers are unprepared for a surrogate decision-making role, because the life expectancy of people with dementia (even advanced dementia) is difficult to predict.[30] In Chinese populations, families avoid mentioning death and are likely to make collective decisions.[31]

The family surrogate decision makers in this study were more likely to prefer no life-sustaining treatment except tube feeding, in contrast to the findings of other studies.[32] This may reflect slowly changing concepts of end-of-life decisions in advanced dementia patients.

Our data also indicate that understanding advanced dementia complications is associated with palliative treatment preferences by family surrogate decision makers, except for tube feeding. Research has demonstrated that nursing home residence and financial burdens influence the life-sustaining treatment decisions of family caregivers for patients with dementia.[33] Information on the poor outcomes of life-sustaining treatment prompts family caregivers to remove these treatments.[31]

We suggest that a lack of knowledge on advanced dementia complications may be a barrier to making palliative care decisions. However, the lower mean complication knowledge score in this study (even though 63% of the caregivers had 12 years of education), show that surrogate decision-makers lack understanding of the clinical complications of advanced dementia. A specific program is critical for both patients with dementia and their families. The contents of this program should include a clinical course on advanced dementia, types of life-sustaining treatment, and life-sustaining treatment outcomes. This may help caregivers to make medical decisions to improve the quality of end-of-life care and reduce incidences of ethical dilemmas.

Several limitations to this study exist. The modest self-disclosure rate reduced the sample size. The majority of the patients having mild to moderate dementia, the outpatient-clinic design, the characteristics of younger age, and the high educational level of the caregivers may restrict the generalization of the findings. The lack of information in this study regarding family caregivers’ religious beliefs limited our analysis of the relationship between disagreement on life-sustaining treatment and religious discordance between family caregiver and demented patients. Because previous studies have suggested that differences in religious beliefs among families can lead to different end-of-life decisions [34], this issue requires further study. The stability of preferences for end-of-life care over time has not been validated in our population, and a cross-sectional assessment cannot capture the dynamic decision-making process.

The family caregivers in this study may have consented to more aggressive treatment for their relatives with dementia than would patients who prefer deciding for themselves. Surrogate decision-making for life-sustaining treatment is related to knowledge on advanced dementia complications, and preference concordance is associated with family caregiver depression. The number of patients with dementia who require end-of-life care is expected to increase rapidly in the future. Understanding the problems that influence family caregiver preferences may help people with dementia and their family caregivers. Supporting caregivers to reduce their distress and levels of depression, and educating them on the clinical features and implications of advanced dementia, may improve palliative care for people with dementia.
